# Enhancer architecture and chromatin accessibility constrain phenotypic space during *Drosophila* development

**DOI:** 10.1016/j.devcel.2022.12.003

**Published:** 2023-01-09

**Authors:** Rafael Galupa, Gilberto Alvarez-Canales, Noa Ottilie Borst, Timothy Fuqua, Lautaro Gandara, Natalia Misunou, Kerstin Richter, Mariana R.P. Alves, Esther Karumbi, Melinda Liu Perkins, Tin Kocijan, Christine A. Rushlow, Justin Crocker

**Affiliations:** 1 European Molecular Biology Laboratory, 69117 Heidelberg, Germany; 2 Department of Biology, New York University, New York, NY 10003, USA

**Keywords:** enhancers, gene regulation, evolution, development, phenotypical novelties, novel expression patterns, reporter assays, random sequences, Drosophila melanogaster, pioneer factors

## Abstract

Developmental enhancers bind transcription factors and dictate patterns of gene expression during development. Their molecular evolution can underlie phenotypical evolution, but the contributions of the evolutionary pathways involved remain little understood. Here, using mutation libraries in *Drosophila melanogaster* embryos, we observed that most point mutations in developmental enhancers led to changes in gene expression levels but rarely resulted in novel expression outside of the native pattern. In contrast, random sequences, often acting as developmental enhancers, drove expression across a range of cell types; random sequences including motifs for transcription factors with pioneer activity acted as enhancers even more frequently. Our findings suggest that the phenotypic landscapes of developmental enhancers are constrained by enhancer architecture and chromatin accessibility. We propose that the evolution of existing enhancers is limited in its capacity to generate novel phenotypes, whereas the activity of *de novo* elements is a primary source of phenotypic novelty.

## Highlights

• Mutation libraries of four developmental enhancers and three sets of random sequences
• Most point mutations rarely result in expression outside of the native pattern
• Random sequences often act as developmental enhancers
• Genomic architecture and accessibility limit developmental enhancers’ evolution

## Graphical abstract

**Figure:**
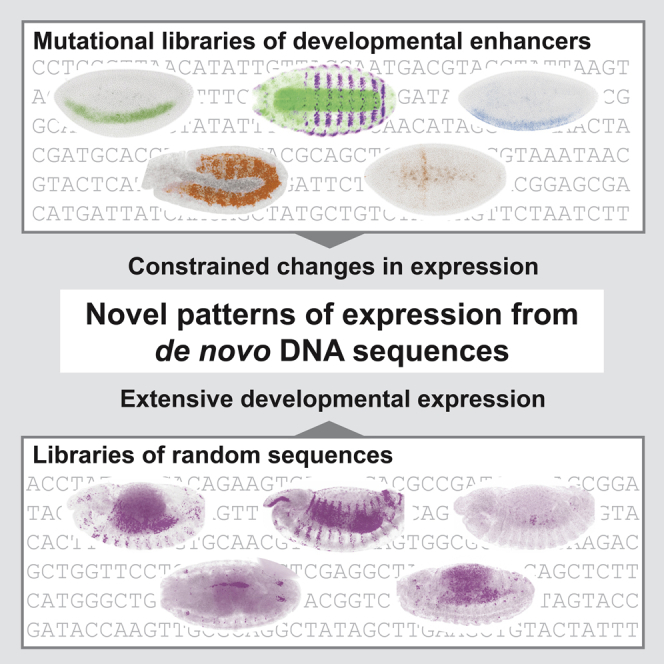

## Teaser

Galupa et al. compared the potential of random sequences versus point mutations of existing enhancers to generate new patterns of expression during *Drosophila melanogaster* development. Their findings point to the activity of *de novo* elements as a primary source for phenotypic novelty in developmental enhancers’ evolution.

## Introduction

Morphological changes generally result from changes in the spatiotemporal regulation of gene expression during development, and thus a major theory in evolutionary developmental biology proposes anatomical evolution to be based on the genetic and molecular mechanisms underlying the evolution of spatial gene regulation.^1^ In line with this, the evolution of *cis*-regulatory elements, such as developmental enhancers,^2^ has been proposed to be a major component of phenotypical evolution across animals.^1^^,^^3^^,^^4^^,^^5^^,^^6^^,^^7^ The so-called “*cis*-regulatory hypothesis” proposes that mutations in enhancers are a common and continuous source of morphological variation, and a means to escape the pleotropic effects of mutations to protein coding regions.^1^^,^^4^ For instance, the evolution of wing pigmentation “spots” in *Drosophila* involved the gain of binding sites for different transcription factors (TFs) in an enhancer controlling a pigmentation gene,^8^ whereas the loss of pelvic structures in stickleback fish occurred via mutations that abrogate the activity of an enhancer controlling the homeobox gene *Pitx1*.^9^ Molecular mechanisms of *cis*-regulatory evolution have also been proposed to include duplications of existing enhancers, *de novo* emergence from existing nonregulatory DNA and co-option or exaptation of transposable elements or enhancers with unrelated activities.^10^^,^^11^^,^^12^^,^^13^^,^^14^^,^^15^^,^^16^^,^^17^^,^^18^

Despite elegant case studies, the extent to which these mechanisms contribute to the regulatory evolution of developmental enhancers remains an open question.^19^^,^^20^ It is still unknown which changes in enhancer function are evolutionarily accessible, or how the distribution of TF binding sites might constrain the evolutionary potential of enhancers.^21^ As such, there is a lack of clarity on the molecular genetic pathways for evolutionary change in animal development based on what is functionally possible versus what is probable and permissible from the standpoint of mutational events and natural selection.^1^

Here, we explored how molecular evolution of existing enhancers versus *de novo* sequences contributes to producing novel patterns of gene expression across *Drosophila melanogaster* embryos. We generated and characterized a panel of unbiased mutation libraries for both classical developmental enhancers and randomly generated sequences; this approach allows to distinguish constraints that emerge from the prior function or evolutionary histories of existing enhancers from constraints that arise from properties of the sequence or locus unrelated to selection processes.

## Results

### Constrained capacity for enhancer-driven expression outside of native expression patterns

**Figure 1:**
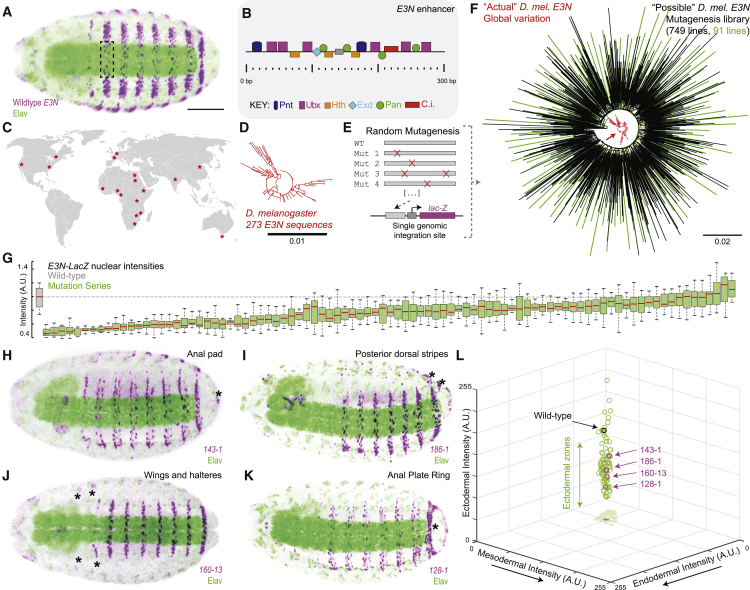
Mutant variants of the *E3N* enhancer have a limited capacity for expression outside native tissues and cell types (A) Pattern of expression driven by wild-type *E3N* at stage 15 (β-galactosidase protein staining). Dashed box demarcates region quantified in (G). Scale bars, 100 μm. (B) Mapped binding site architecture for *E3N*. (C) Collection locations of sequenced *Drosophila melanogaster* strains.^97^ (D) Phylogenetic tree of *E3N* sequences across *D. melanogaster* strains. Units of the scale bar are units of substitutions per site of the sequence alignment. (E) Schematic of enhancer variants and reporter gene construct used for integration into the *D. melanogaster* genome. (F) Phylogenetic tree of *E3N* sequences across *D. melanogaster* strains (red) and of *E3N* sequences from our mutational library (black and green; in green, 91 lines selected for further characterization). Units of the scale bar are units of substitutions per site of the sequence alignment. (G) Nuclear intensities of the A2 segment region (see region demarcated in A) across 91 lines, normalized to wild-type *E3N* (n = 10 embryos per line). a.u., arbitrary units of fluorescence intensity. (H–K) Examples of mutant variants leading to reporter expression outside the wild-type *E3N* pattern. In (H) and (I), the expression associated to esophagus is likely an artifact of the construct used, as observed in other lines unrelated to *E3N*. (L) 3D plot showing fluorescence intensities for 91 lines across three regions of the embryo with different germ-layer origins (see Figures S1G and S1H). Each dot corresponds to the average value for one variant enhancer line.

We first set out to investigate whether and how mutations across developmental enhancers could lead to ectopic, novel expression patterns. We have previously generated a mutation library for the *E3N* enhancer (292 bp), which regulates the expression of *shavenbaby* (*svb*; Figures 1A and 1B).^21^ This mutation library included 749 variants and most mutations led to changes in transcriptional outputs (e.g., levels and location).^21^ This library represents a ∼6 times larger sequence space than the natural variation found for *D. melanogaster E3N* from samples across the world (Figures 1C–1F and S1A). To investigate novel expression patterns, we selected a subset of lines harboring 1–10 point mutations for further characterization (see STAR Methods for further details); these lines come from different regions of the sequence space covered by the total library (Figure 1F; Table S1A) and showed a spectrum of effects in terms of expression levels (Figure 1G). We found that 22% of the lines showed expression outside of the usual *E3N-*driven ventral stripes, in regions such as prospective anal pads, wing and haltere imaginal discs and other structures (Figures 1H–1K). However, these regions are ectopic regions for the enhancer but not for the target gene—they correspond to ectoderm-derived regions where *svb* is expressed.^22^^,^^23^

To evaluate ectopic expression across regions derived from different germ layers, we quantified reporter expression intensity in the selected lines (Figures S1G and S1H) and detected no expression in regions derived from germ layers other than the ectoderm (Figure 1L), whereas variable levels of expression along the “ectoderm” axis could be seen (Figure 1L). These results suggest that evolving new patterns of expression upon point mutations of a developmental enhancer is possible but developmentally biased to specific lineages.

### The emergence of ectopic expression patterns upon mutagenesis of developmental enhancers is rare

**Figure 2:**
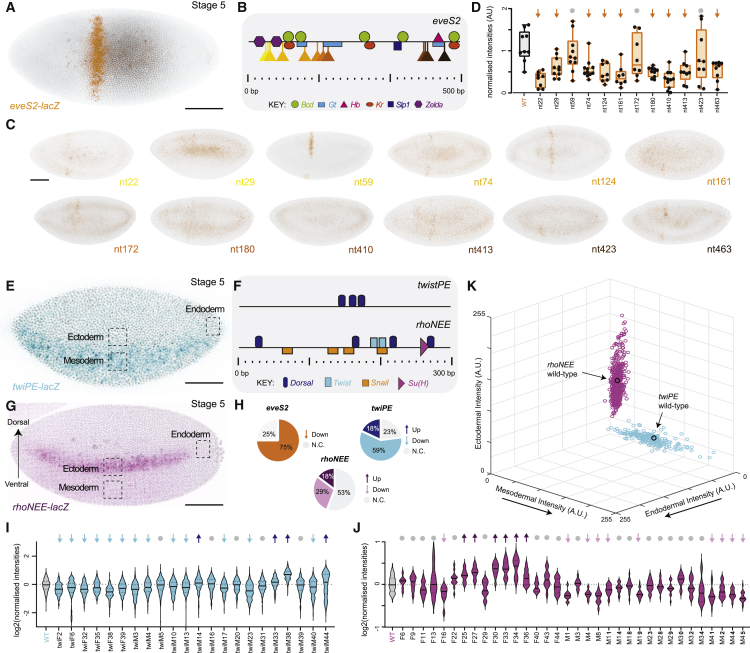
Mutagenesis across early developmental enhancers alters gene expression only within native patterns of expression (A) Pattern of expression driven by wild-type *eveS2* at stage 5 (lacZ mRNA staining). Scale bars, 100 μm. (B) Known binding site architecture for *eveS2*. Location of point mutations is indicated. (C) Examples of stained embryos from different *eveS2* single-nucleotide mutant variants. The name of each line corresponds to the location of the point mutation (compare with B). (D) Fluorescence intensities of the region where the wild-type *eveS2* shows a stripe across 12 single-nucleotide *eveS2* variants (n = 8–11 embryos per line). Lines marked with an arrow are statistically significantly different from wild type (p < 0.05; two-tailed t test). a.u., arbitrary units of fluorescence intensity. (E) Pattern of expression driven by wild-type *twiPE* at stage 5 (lacZ mRNA staining). (F) Known binding site architecture for *twiPE* and *rhoNEE*. (G) Pattern of expression driven by wild-type *rhoNEE* at stage 5 (lacZ mRNA staining). (H) Summary of changes in expression levels for the *eveS2*, *twiPE*, and *rhoNEE* lines. (I and J) Nuclear intensities across *twiPE* (I) and rhoNEE (J) variants (n = 6–27 embryos per line). Lines marked with an arrow (up or down) are statistically significant from wild type (p < 0.05; two-tailed t test). (K) 3D plot showing fluorescence intensities for *twiPE* (blue) and *rhoNEE* (purple) lines across three regions of the embryo illustrated in (I) and (J). Each dot corresponds to one embryo; three embryos per line were quantified.

The hierarchical position of an enhancer in its gene regulatory network(s) is expected to influence the effects of its loss or redeployment;^12^ enhancers at *higher* positions in the network are expected to be more robust, given that mutations affecting them would have more pleiotropic effects. We thus decided to analyze additional “classical” enhancers involved in early development (*higher* in the network) to explore whether the transcriptional constraints we observed for *E3N* mutagenesis are a general property of developmental enhancers, or linked to the fact that *E3N* regulates a terminal selector gene (*lower* in the network) in later development.^24^ The “early” enhancers we explored include *eveS2* (484 bp), important for anterior-posterior specification (Figures 2A and 2B),^25^^,^^26^^,^^27^ and *rhoNEE* and *twiPE* (359 and 290 bp, respectively), both involved in dorsoventral patterning (Figures 2E–2G), in the neurogenic ectoderm and mesoderm, respectively.^28^^,^^29^^,^^30^^,^^31^^,^^32^^,^^33^ For each of these enhancers, we generated mutant libraries using the same setup as for the *E3N* library:^21^ each variant was cloned upstream of a heterologous *hsp70* promoter driving *lacZ* reporter expression and integrated into the *Drosophila* genome at a specific landing site, amenable to expression across different tissues and stages (Figures S1B–S1F). Using a PCR error-rate of ∼0.5% per molecule, we isolated enhancer variants containing approximately 1–5 mutations in 12–36 independent fly lines per enhancer (Table S1).

We examined reporter activity across all lines in the early embryo (stage 5) and found similar trends for all of them. On the one hand, mutations often led to significant changes in expression levels, and on the other hand, changes in expression were restricted to the native pattern—no ectopic expression was observed. For *eveS2* (Figure 2A), each variant contained a single mutation only, almost none overlapping a known binding site (Figures 2B and 2C). Yet, 75% led to significantly reduced expression compared with control (Figures 2D and 2H), suggesting that it is relatively easy to “break” the minimal eveS2 enhancer, consistent with unsuccessful attempts to build this enhancer *de novo*.^34^^,^^35^ In no case did we observe expression outside of the eve stripe 2 region. Similar results were found for *rhoNEE* and *twiPE*: 47% and 77% of enhancer variants, respectively, showed statistically significant changes in nuclear intensities compared with control (Figure 2H); for *rhoNEE*, 18% showed higher expression and 29% showed lower expression (Figure 2J); for *twiPE*, these values were 18% and 59% respectively (Figure 2I). These effects did not seem to correlate with the number of mutations per enhancer (Figure S2A) nor with the length of the enhancer (compare Figures 2B and 2F with 2H). Again, despite clear changes in levels for most mutant variants, we noted that expression outside of the typical area of expression for each enhancer was never observed—quantification of expression in control and mutant lines across regions of the embryo that will give rise to ectoderm (lateral region of the embryo), endoderm (posterior region of the embryo), and mesoderm (ventral region of the embryo; regions highlighted in Figures 2E and 2G) revealed that mutant lines showed changed levels of expression but always within the ectoderm and “mesoderm” regions only, for *rhoNEE* and *twiPE* enhancers, respectively (Figure 2K). In summary, most mutations led to changes in expression levels within native zones of expression; thus, the results suggest that the “molecular evolution” by point mutations of developmental enhancers is not likely to result in novel expression patterns.

**Figure 3:**
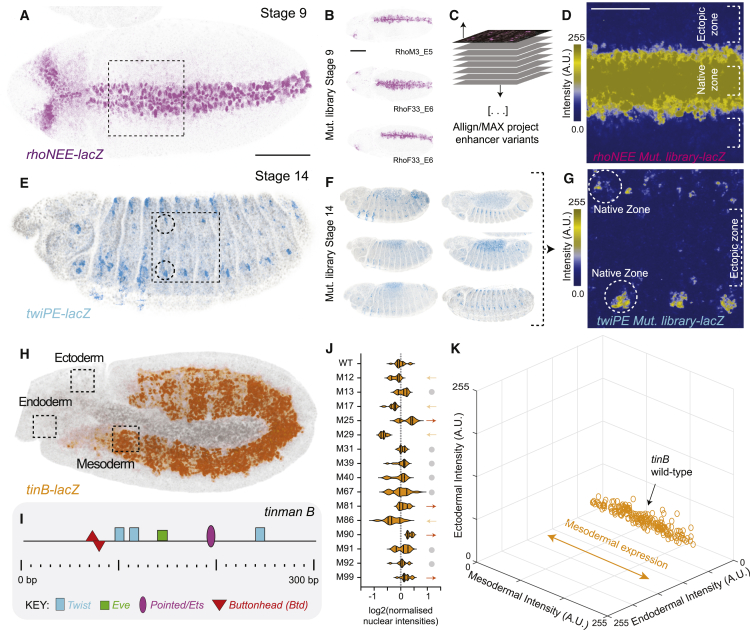
Mutagenesis across late developmental enhancers alters gene expression only within native patterns of expression (A) Pattern of expression driven by wild-type *rhoNEE* at stage 9 (β-galactosidase protein staining). Scale bars, 100 μm. (B) Examples of stained embryos from different *rhoNEE* mutant variants. Scale bars, 100 μm. (C) Schematic of alignment and overlaying of individual Z projections of maximum intensity for *rhoNEE* mutant variants. (D) Heatmap of aggregated Z projections. Scale bars, 50 μm. (E) Pattern of expression driven by wild-type *twiPE* at stage 14 (β-galactosidase protein staining). (F) Examples of stained embryos from different *twiPE* mutant variants. (G) Heatmap of aggregated Z projections upon alignment of individual Z projections of maximum intensity for *twiPE* mutant variants. (H) Pattern of expression driven by wild-type *tinB* at stage 10 (β-galactosidase protein staining). (I) Known binding site architecture for *tinB*. (J) Nuclear intensities across *tinB* variants (n = 10–18 embryos per line). Lines marked with an arrow (up or down) are statistically significant from wild type (p < 0.05; two-tailed t test). (K) 3D plot showing fluorescence intensities for *tinB* lines across three regions of the embryo as illustrated in (H). Each dot corresponds to one embryo; at least ten embryos per line were quantified.

Considering that such pleiotropic effects could be revealed throughout development,^22^ we analyzed expression in embryos at later stages (stage 9 and 14) for the *rhoNEE* (Figures 3A and S2B) and *twiPE* libraries (Figure 3E and S2C), but we observed no ectopic expression in the mutant lines compared with the control (Figures 3B–3D, 3F, and 3G). We also generated an additional mutational library for *tinB*, a 411-bp enhancer that controls a mesoderm-specific gene throughout a broad developmental window (Figures 3H and 3I; Table S1).^36^^,^^37^ Similar to what we found for early enhancers, 47% of enhancer variants showed significant changes in enhancer activity (Figures 3J and S2D; 20% showed increased expression, 27% showed decreased expression), yet no ectopic expression was observed (Figure 3K).

Finally, we tested whether ectopic expression could be “forced” upon recruitment of a ubiquitously expressed synthetic TF. The *rhoNEE* enhancer has been previously engineered to contain binding sites for a transcription activator-like effector (TALE) DNA-binding protein.^38^ We crossed fly lines harboring *rhoNEE* enhancers with one, two, or three TALE-binding sites with a line containing a TALE protein fused to the strong activation domain VP64^39^ and expressed via an ubiquitous nos::Gal4 driver, and quantified expression across different regions of the early embryo (Figure S2E). The higher the number of binding sites for the synthetic TF, the higher the expression within the usual regions of *rhoNEE* expression. However, it was not until there were two or more binding sites (16-bp long) that appreciable expression was generated outside of the native zones of expression (Figure S2E). Together, these results reveal that the *rhoNEE* enhancer is not “intrinsically” refractory to expression outside of its usual pattern of expression but rather requires a considerably larger recruitment of activators to the locus. The fact that we do not observe ectopic expression in the enhancer libraries analyzed suggests that evolutionary constraints are imposed on developmental enhancers.

### Random sequences lead to extensive expression across developmental time and space

**Figure 4:**
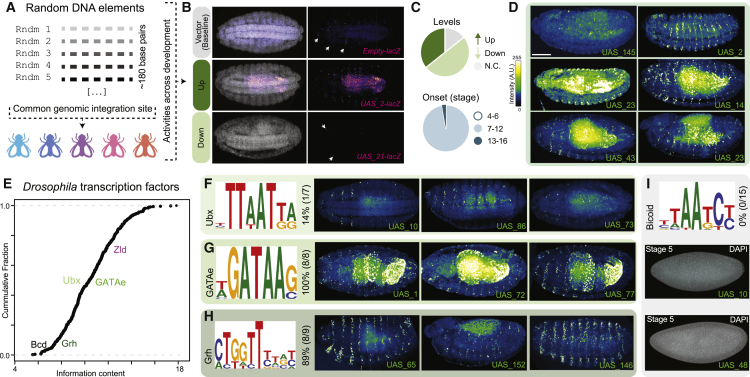
Random DNA sequences often drive reporter expression during development (A) Schematic of the UAS library. (B) Expression patterns at stage 15 were compared with the reporter with no sequence cloned upstream (top) and classified as “up” (middle) or “down” (bottom), depending on whether expression was increased or decreased, respectively. (C) Summary of changes in expression levels at stage 15 (top) based on panel (B), and of developmental period in which expression is first observed (bottom). (D) Examples of stained embryos from different random DNA sequences. (E) Cumulative distribution function of the expected frequency of *Drosophila* TF motifs in random DNA. (F) Ubx motif, percentage of lines showing expression among random DNA lines with a Ubx motif and examples of corresponding embryos. (G) GATA motif, percentage of lines showing expression among random DNA lines with a GATA motif and examples of corresponding embryos. (H) Grh motif, percentage of lines showing expression among random DNA lines with a Grh motif and examples of corresponding embryos. (I) Bicoid motif, percentage of lines showing expression among random DNA lines with a Bcd motif and examples of corresponding embryos.

We interrogated the extent to which *de novo* sequences, devoid of evolutionary constraints, could act as enhancers and drive expression across the embryo and across development. We synthesized random sequences (∼180 bp), inserted them upstream of *hsp70* promoter driving *lacZ* (similarly to the enhancer libraries) and integrated them into the fly genome at the same genomic location (Figures 4A and S3A). These sequences included a motif (UAS) for the yeast Gal4 TF,^40^^,^^41^ which is not present in the fly and thus, this motif should be “neutral”; this design was chosen so that these sequences have a comparable architecture to libraries containing other motifs (see later). We isolated 56 fly lines harboring unique sequences (Table S1), for which we stained embryos at different stages to determine reporter gene’s expression pattern(s). Surprisingly, 86% of sequences led to changes in reporter expression at least in some cells and/or at some developmental stage, compared with expression of the reporter with no sequence cloned upstream (Figures 4B–4D and S3B). The other surprising observation was that despite such pervasive expression, we never observed expression in the early embryo (Figure 4C). Given the variable consensus sites found in multicellular systems, such libraries are expected to have a range of motifs with variable information content^42^^,^^43^ (Figure 4E). To explore the expression patterns observed, we conducted motif searches across all random sequences for *Drosophila* developmental TFs (Figure 4E; STAR Methods). Motifs found included Ultrabithorax (Ubx), GATA, Grainyhead (Grh), and Bicoid (Bcd) motifs (Figures 4F–4I). Interestingly, 100% or 80% of the random DNA elements containing a GATA or Grh motif, respectively, showed expression (Figures 4G and 4H), consistent with their previously reported predictive power^44^^,^^45^ and with the expression patterns of the respective TFs. In contrast, only 14% of elements with a Ubx motif showed expression (Figure 4F), and none of the elements containing a Bcd motif showed expression (Figure 4I), consistent with the absence of expression in the early embryo for all random sequences. We calculated whether our random sequences were biased for motifs of late-development TFs, but this did not explain the absence of early expression (average per sequence: ∼3.9 hits per early-specific motif versus ∼3.4 hits per late-specific motif; see STAR Methods).

### Specific motifs can potentiate emergence of enhancer activity

**Figure 5:**
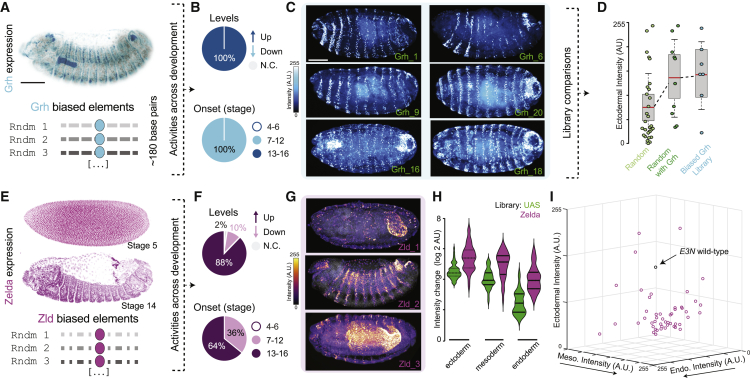
Specific DNA motifs enhance likelihood of reporter expression during development (A) Staining for Grh transcription factor (top) and schematic of the Grh-library (bottom). (B) Summary of changes in expression levels (top) compared with the reporter with no sequence cloned upstream (Figure 4B) and of developmental period in which expression is first observed (bottom). (C) Examples of stained embryos from different Grh-biased sequences. (D) Quantification of fluorescent intensities in ectoderm-associated region for all random DNA sequences, for random DNA sequences with Grh motifs (subset of all random DNA sequences) and for Grh-biased sequences. (E) Staining for Zld transcription factor (top) and schematic of the Zld-library (bottom). (F) Summary of changes in expression levels at stage 15 (top) compared with the reporter with no sequence cloned upstream (Figure 4B) and of developmental period in which expression is first observed (bottom). (G) Examples of stained embryos from different Zld-biased sequences. (H) Quantification of fluorescent intensities for Zld-biased lines across three regions of the embryo (see Figures S1G and S1H). (I) 3D plot showing fluorescence intensities for Zld-biased lines, based on (H). Each dot corresponds to one line. For reference, fluorescence intensity for the wild-type *E3N* sequence is shown (from Figure 1L).

Completely random sequences thus seem to have a high potential of driving expression, and this can be associated to particular motifs. Given the association between chromatin accessibility and transcriptional permissiveness,^46^ as well as studies suggesting that chromatin accessibility might underlie enhancer evolution,^47^^,^^48^ we generated “biased” random libraries in which we included a Grh motif (Figure 5A; 7 lines, Table S1) or a Zelda motif (Figure 5E; 41 lines; Table S1) approximately at the center of random sequences. Grh and Zelda are TFs in the fly reported to have “pioneer activity”^49^^,^^50^—their binding is associated with “opening” chromatin, rendering enhancers more accessible to binding by other TFs.^51^^,^^52^^,^^53^^,^^54^^,^^55^^,^^56^^,^^57^^,^^58^ Though Zelda is usually associated with early fly development, it is expressed throughout development (Figure S4A), and its late embryonic knockout has phenotypical consequences (Figure S4B). Consistent with the idea of pioneer activity, an even higher proportion of random sequences from the Grh and Zld-biased libraries drove expression compared with the UAS library (Figures 5B, 5C, 5F, 5G, and S4C). Not only a higher number of lines was associated with expression for the biased libraries, but also expression levels were higher when compared with the UAS library, regardless of the region of the embryo (Figures 5D, 5H, and 5I). To further test the potential of these motifs, we added one or two Zelda motifs to the developmental enhancers we tested initially (*eveS2*, *rhoNEE*, *twiPE*, and *tinB*) and found a significant increase in reporter expression levels for all enhancers within their native patterns of expression (Figures S5A–S5H). For the *eveS2* lines, we additionally observed novel, ectopic expression (Figures S5A–S5H), suggesting that the Zelda motifs might “unlock” cryptic sites contained in *eveS2*. We tested whether *eveS2* contained more predicted motifs than the other enhancers, but we did not find any significant differences in the number of hits (0.07 for *eveS2* versus 0.10, 0.12, and 0.05 for *rhoNEE*, *tinB*, and *twiPE*, respectively; normalized per enhancer length).

To explore the possibility that the occurrence of specific motifs throughout the genome might contribute to the emergence of (*de novo*) enhancers, we selected genomic sequences containing high-affinity Ubx/Hth motifs (ATGATTTATGAC)^59^ present in *D. melanogaster* but not in other *Drosophila* species (Figures S5I–S5L). Such motifs have been demonstrated to augment chromatin accessibility^60^ and are broadly used across development, providing a counterpoint to our synthetic libraries. Strikingly, when we tested their enhancer potential with the *lacZ* reporter assay, all sequences showed enhancer activity (Figures S5I–S5L). Mutating the Ubx/Hth motif in each of those sequences led to a dramatic reduction in expression for six out of seven of those sequences (Figures S5I–S5L), indicating that these motifs clearly have the capacity to drive expression across development. These results support the idea that specific sequence motifs might prime genomic sequences to act and/or evolve as enhancers.

## Discussion

We used transgenesis-based mutagenesis and *de novo* gene synthesis during fly embryogenesis to investigate evolutionary pathways for enhancer activity. We used fly development to explore how novel patterns of gene expression might appear from either molecular evolution of developmental enhancers or random sequences. Notably, while reporter gene assays and minimal enhancers may not reflect the full regulatory activities of native loci,^61^^,^^62^^,^^63^ such an approach allows us to evaluate a broad range of “possible” enhancer variation in a controlled experimental setup, without associated fitness costs and allowing a broader exploration of evolution and development without the complexities and historical contingencies found in nature. Furthermore, using such an assay in a developmental model system, which generates an embryo in 24 h, we can assay regulatory activities across ∼100,000 cells of different lineage origins.^64^

Using this approach, we found that most mutations in enhancers led to changes in levels of reporter gene expression, but almost entirely within their native zones of expression (Figures 1, 2, and 3), similar to previous studies using transgenic mutagenesis of the *Shh* enhancer in murine embryos,^65^ or the *E3N* enhancer^21^ and the wing *spot*^*196*^ enhancer^66^ in fly embryos. Consistent with our results, known phenotypic evolution through nucleotide mutations of standing regulatory elements seems to appear either through changes in the levels or timings of expression within native zones or the loss of regulatory activities. For example, the evolution of pigmentation spots in fly wings occurred via a specific spatial increase in the melanic protein Yellow, which is uniformly expressed at low levels throughout the developing wings of fruit flies;^8^ see Frankel et al.^67^ and Rebeiz et al.^68^ for other examples of evolution within native patterns of expression. Evolution of other traits such as thoracic ribs in vertebrates,^69^ limbs in snakes,^70^ pelvic structures in sticklebacks,^9^ and seed shattering in rice^71^ are all associated with loss of enhancer activity due to internal enhancer mutations. Additionally, mutations have been found to occur less often in functionally constrained regions of the genome, suggesting that mutation bias may reduce the occurrence of deleterious mutations in regulatory regions.^72^

Consistent with these results, phenotypic novelties underlain by enhancer-associated ectopic gains of expression are reportedly due to transposon mobilization,^73^^,^^74^^,^^75^^,^^76^ rearrangements in chromosome topology^77^^,^^78^^,^^79^ or *de novo* evolution of enhancers from DNA sequences with unrelated or nonregulatory activities.^13^^,^^17^^,^^19^^,^^80^^,^^81^^,^^82^^,^^83^ Previous studies have explored the potential of random DNA sequences to lead to reporter gene expression, either as enhancers or promoters, especially in cell lines of prokaryotic or eukaryotic origin.^45^^,^^84^^,^^85^ These have shown that there is a short (or sometimes null) mutational distance between random sequences and active *cis*-regulatory elements,^85^ which may improve evolvability. In our study, we tested random sequences in a developmental context and found that most showed enhancer activity across several types of tissues and developmental stages (Figure 4). These results are consistent with a study that tested enhancer activity of all 6-mers in developing zebrafish embryos and found a diverse range of expression for ∼38% of the sequences at two developmental stages.^20^ We observed expression driven by random sequences even in the absence of motifs within their sequence for TFs with pioneering activity (Figure 4). Yet, when such motifs were included, nearly all sequences acted as “strong” enhancers (leading to high levels of expression) (Figure 5), consistent with the “evolutionary barrier” to the formation of a novel enhancer being lower in regions that already contain motifs for DNA-binding factors, which can “act cooperatively with newly emerging sites.^11^”

It is interesting to note that, despite the high potential of random sequences to be expressed during development and across cell types, we never observed expression prior to gastrulation; this was not evaluated in the zebrafish study or in other studies. This may be due to the rapid rates of early fruit fly development, in which gene expression patterns are highly dynamic, and cell-fate specifications occur within minutes.^86^ As such, there may be extensive regulatory demands placed on transcriptional enhancers, reflected in the clusters of high-affinity binding sites common across early embryonic developmental enhancers^87^ as well as their extensive conservation in function^88^ and location.^89^ In the future, it will be interesting to explore how regulatory demands that change across development—such as nuclear differentiation, network cross-talk, and metabolic changes— are reflected in regulatory architectures and their evolvability.

The observation that most random sequences led to expression suggests that the potential of any sequence within the genome to drive expression is enormous and thus “an important playground for creating new regulatory variability and evolutionary innovation.^80^” This was further supported by the regulatory potential of the genomic sequences we tested, containing Ubx/Hth motifs; indeed, the results from our work imply that enhancers would more likely evolve from sequences that contain or are biased toward specific motifs (e.g., GATA and Zelda). Perhaps the challenge from an evolutionary perspective has not been what allows expression, but what prevents expression; thus, mechanisms that repress “spurious” expression might have evolved across genomes. This is in line with propositions that nucleosomal DNA in eukaryotes has evolved to repress transcription,^90^^,^^91^ along with transcriptional repressors and other mechanisms such as DNA methylation, as a response (at least partially) to “the unbearable ease of expression” present in prokaryotes.^92^ The action of such repressive mechanisms could also explain why mutagenesis of developmental enhancers, which are subject to evolutionary selection, does not easily lead to expression outside their native patterns of expression. In sum, our findings raise exciting questions about the evolution of enhancers and the emergence of novel patterns of expression that may underlie new phenotypes, suggesting an underappreciated role for *de novo* evolution of enhancers by happenstance. Genetic theories of morphological evolution will benefit from comparing controlled, multi-dimensional laboratory experiments with standing variation;^93^ such an integrative approach could provide the frameworks that will enable us to make both transcriptional and evolutionary predictions.

### Limitations of the study

One limitation of our study lies on the numbers—we have tested a significant number of enhancer variants, but it is still possible that we would have captured ectopic expression more frequently had we tested a larger set of enhancer variants. Also, in principle, a higher number of mutations per enhancer could have also enhanced the likelihood of ectopic expression. Previous work from our lab with the E3N enhancer reported that indeed the proportion of lines with ectopic expression increased with the number of mutations.^21^ However, this increase plateaued around 20%–30% for lines with ∼3+ mutations per enhancer and in this study, the number of mutations in the enhancer variants for *twiPE*, *rhoNEE*, and *tinB* ranges from 1 to 7 mutations, so we could have expected to capture a number of lines with ectopic expression. Importantly, our assay captures millions of years of variation in a controlled setting decoupled from fitness costs. It is also possible that ectopic expression might be present in developmental stages that we have not analyzed. Finally, would the results be different if we had used a different promoter? We have not tested this formally, but based on published literature, we believe that using a different promoter would not have major implications in the results observed. Testing a total of 27 enhancer-promoter combinations in human cells, efficiency of enhancers has been shown to be approximately the same irrespective of the type of promoter used,^94^ and a recent combinatorial analysis of 1,000 human promoters and 1,000 human enhancers confirmed that most enhancers activate all promoters by similar amounts.^95^ These studies, in cell lines, could only address levels of expression, not spatial patterns—but very recently published results from the lab^96^ show that developmental promoters in fly embryos can drive a range of outputs but do not affect spatial aspects of expression, only levels.

## STAR★Methods

### Key resources table

**Table:** 

REAGENT or RESOURCE	SOURCE	IDENTIFIER
**Antibodies**		

Mouse anti-betagalactosidase	Promega	Z378; RRID: AB_2313752
Donkey anti-mouse AlexaFluor 555	ThermoFisher	A31570; RRID: AB_2536180
Sheep anti-digoxigenin	Roche	11333089001; RRID: AB_514496
Rabbit anti-FITC	ThermoFisher	A889; RRID: AB_221561
Donkey anti-rabbit AlexaFluor 488	ThermoFisher	A21206; RRID: AB_2535792
Donkey anti-sheep AlexaFluor 555	ThermoFisher	A21436; RRID: AB_2535857

**Chemicals, peptides, and recombinant proteins**		

Blocking solution	Roche	11921673001
In-vitro transcription labeling kit	Roche	11175025910
Prolong Gold with DAPI	ThermoFisher	P36935
Paraformaldehyde ampules (10x 10ml)	EMS Diasum	15710
NaCl	Merck	1064041000
TritonX-100	Sigma	T9284
EGTA	Sigma	E3889
Heptane	Sigma	246654-1L
Heparin	Sigma	H3393-100KU
Methanol	Merck	1060091000
Formamide	Sigma	47671-1L-F
SSC 20x	Sigma	SRE0068-1L
Salmon Sperm DNA Solution UltraPure	ThermoFisher	15632011
Tween-20	Sigma	P9416
Ethanol	Merck	1009831000
Xylenes	VWR	ACRO422685000

**Experimental models: Organisms/strains**		

attP2 line	Bloomington Drosophila Stock Center	25710
VK33 line	Bloomington Drosophila Stock Center	32543
w1118 line	Bloomington Drosophila Stock Center	5905

### Resource availability

#### Lead contact

Further information and requests for resources and reagents should be directed to and will be fulfilled by the lead contact, Justin Crocker (justin.crocker@embl.de).

#### Materials availability

Plasmids, DNA libraries and fly lines generated in this study will be made available from lead contact upon request. A mutual transfer agreement might have to be signed for some materials.

### Experimental model and subject details

The experimental model in this study is *Drosophila melanogaster*. All transgenic lines generated in this study were based on lines attP2 (Bloomington Stock Number: 25710) and VK33 (Bloomington Stock Number: 32543). When outcrosses were needed, we used line w1118 (Bloomington Stock Number: 5905). Information on developmental stages used is described in the main text, figures and/or method details (see below). All fly lines were kept in mite-free conditions, grown at room temperature in the lab or in a room where temperature is set at 25°C, in plastic vials or plastic bottles supplemented with fly food (1.8% m/v yeast, 1% m/v soy flour, 8% m/v cornmeal, 8% m/v malt extract, 1.2% m/v agar, 2.2% m/v sugar beet syrup, 0.625% v/v propionic acid, 1.2% v/v nipagin 20% in ethanol). All fly lines were flipped to new vials/bottles at least every two weeks.

### Method details

#### Fly strains and constructs

##### E3N library

We categorized the 749 variants from the original *E3N* library^21^ by the number of mutations each variant contained. Using python's random module, we randomly selected 10 lines from the single mutation bin to the 10 mutations bin (selection without replacement, 100 lines total). If the line had been lost, we re-ran the generator to select a replacement line. Each line was fixed, stained, and imaged with a control *E3N* WT. If there were less than 10 embryos in the correct orientation and developmental stage on the microscope slide, the line was removed from the analysis. In the end, we had 10 lines with one mutation, 9 with two mutations, 9 with three mutations, 9 with four mutations, 7 with five mutations, 9 with six mutations, 9 with seven mutations, 10 with eight mutations, 8 with nine mutations, and 10 with ten mutations (please see Table S1 for details). Thus, 505 total mutations were tested in 91 mutants, with the average number of mutations in this dataset ∼5.5 (505/91). Sequences of the selected lines are provided in Table S1.

##### All other libraries

All mutant and random enhancer sequences were synthesized and cloned (GenScript) into pLacZattB plasmid at HindIII/XbaI site (see more details below about the synthesis of random sequences). *eveS2*-related lines were injected into attP2 line, all other constructs were injected into VK33 line; injections done by Genetivision. Transgenic lines were homozygosed and genotyped; sequences are listed in Table S1.

##### Synthesis of random sequences

Random sequences were synthesised by GenScript using their Precision Mutant Libraries service. Thousands of random DNA variants were synthesised, with no selection requirements other than size (chosen to be ∼180bp, which was at the time the maximum size that their technology allowed). More specifically, 70-100bp random sequences were synthesised as oligo pools flanked on one side by either HindIII or XbaI sites, and on the other side by a partial motif for BsaI (a TIIS restriction endonuclease). These random sequences and the backbone plasmid (pLacZattB) were digested with HindIII, XbaI and/or BsaI, and digested fragments were then mixed and assembled to the final plasmid library. The structure of the final library is [backbone-HindIII-random-TIIS-random-XbaI-backbone].

#### Embryos collection and fixation

Flies were loaded into egg collection chambers, left to acclimatize for 3-4 days and then embryos were collected for either four or sixteen hours, for early and late stages, respectively. Embryos were dechorionated in 5% bleach for 2min, abundantly rinsed with water and washed in a saline solution (0.1 M NaCl and 0.04% Triton X-100), before transfer to scintillation vials containing fixative solution (700 μl 16% PFA, 1.7 ml PBS/EGTA, 3.0 ml 100% heptane). Embryos were fixed for 25 min, shaking at 250 rpm. The lower phase was then removed, 4.6 mL 100% methanol added and vials vortexed at maximum speed for 1min. The interphase and upper phase were removed and the embryos were washed thrice in fresh methanol. Embryos were stored at -20 °C until processed.

#### Reporter gene expression analysis

##### *In situ* hybridization (probes)

probes for *lacZ* (reporter) and *snail* (internal control) were generated from PCR products using the in vitro transcription (IVT) kit from Roche (#11175025910) and following manufacturer’s instructions. A list of primer sequences for each PCR product can be found in Table S1. For each gene, distinct PCR products were pooled before IVT reaction. Probes were diluted in hybridization buffer (Hyb; 50% formamide, 4X SSC, 100 μg/mL salmon DNA, 50 μg/mL heparin, 0.1% Tween-20) at 50ng/μL. Prior to hybridization, a probe solution was prepared (per sample, 50 ng of each probe in 100 μL), denatured at 80 °C for 5min, then immediately put on ice for 5min, and finally incubated at 56 °C for 10min before added to the embryos.

##### *In situ* hybridization (procedure)

embryos stored in methanol were washed in methanol/ethanol (50:50), three-times in 100% ethanol and then permeabilized in xylenes (90% in ethanol) for 1h, after which embryos were washed six times in ethanol and three times in methanol. Embryos were then washed three times in PBT (PBS + 0.1% Tween-20) before post-fixation for 25min in fixative solution (225 μl 16% PFA, 500 μl PBT). Embryos were then washed several times in PBT for 40 min, followed by a wash in PBT/Hyb (50:50) at room temperature and a 30min-wash in pre-warmed Hyb at 56 °C. Embryos were then incubated with probe solution at 56°C overnight. The next day, embryos were washed in Hyb (three quick washes followed by three 30-min washes), then in Hyb/PBT (50:50), then in PBT several times for one hour before incubated for 30 min in blocking solution (Roche #11921673001; diluted 1:5 in PBT). Embryos were then incubated in blocking + primary antibodies diluted 1:500 (anti-DIG, Roche #11333089001; anti-FITC, ThermoFisher #A889) at 4 °C overnight. The next day, embryos were washed in PBT (three quick washes followed by four 15-min washes), and then incubated at room temperature in blocking solution + secondary antibodies diluted 1:500 (AlexaFluor 488 and 555, ThermoFisher #A21206 and #A21436, respectively). After 2 hours, embryos were washed in PBT (three quick washes followed by four 15-min washes), mounted on Prolong Gold with DAPI (ThermoFisher, P36935) and left to curate overnight before imaging.

##### Immunofluorescence

embryos stored in methanol were washed in PBT (three quick washes followed by four 15-min washes), then in blocking solution for 30 min (Roche #11921673001; diluted 1:5 in PBT), before incubated overnight at 4 °C in blocking solution + primary antibody diluted 1:500 (mouse anti-betagalactosidase, Promega #Z378). The next day, embryos were washed in PBT (three quick washes followed by four 15-min washes), and then incubated at room temperature in blocking solution + secondary antibody (donkey anti-mouse AlexaFluor 555, ThermoFisher #A31570). After 2 hours, embryos were washed in PBT (three quick washes followed by four 15-min washes), mounted on Prolong Gold with DAPI (ThermoFisher, P36935) and left to curate overnight before imaging.

##### Microscopy and data analysis

embryos were imaged using a confocal microscope Zeiss LSM 880 confocal. Images were processed using a combination of automated scripts with manual curation. For 3D plots showing signal intensity across three regions of the embryo (Figures 1l, 2K, 3G, and 5I), images were analyzed in ImageJ: a circular ROI of constant size was used to measure average intensity across the different regions (selected as shown in figures); a co-stain for *snail* was used to help demarcate the presumptive germ layers. The number of lines/embryos analyzed for each case are indicated in figure legends. For analyzing *E3N* mutant lines,^98^ individual nuclei were identified using the automated threshold algorithm on ImageJ and a watershed to split large ROIs; average intensities for each nucleus were measured. For analyzing *eveS2* mutant lines, we used ImageJ to perform Z-projections of max intensity, and a MATLAB (version R2018b; The MathWorks, Inc.) automated image analysis pipeline (Data S1) was developed to capture expression signal along the AP axis on stage 5 embryos. For automated rotation, an ellipse was fitted on a masked embryo, and embryos were rotated based on the maximum Feret diameter. For quantification, a section with 30% of the height of the embryo was taken at a middle position and along the AP axis of each embryo. From this image section, the intensities from all the rows in the image matrix were averaged for each pixel position along the AP axis. The integration and analysis from each of these resultant AP embryo expression profiles were done in R.^99^ These expression profiles were smoothed with a Gaussian filter and then a linear interpolation was performed in order to have fixed samples number for the AP axis. Background removal and normalization were done based on the 10% and 50% quantile intensities, respectively, from the last 20% of the egg length. All embryos expression profiles per each genetic line were bootstrapped in order to see their reporter expression distribution along the AP axis. The bootstrapping was done using a confidence interval of 95% with 1000 replicates. For analyzing *twiPE* mutant lines, we used ImageJ to perform background subtraction from Z-projections of max intensity, rotate embryos to a vertical position and select a ROI at a defined position based on the intersection between 50% of the embryo long axis and the border of the *snail* RNA signal. We then used MorphoLibJ plugin in ImageJ to mask nuclei (volume higher than 3) and extracted intensities. For analyzing *rhoNEE* mutant lines, we used a custom code written in MATLAB (version R2018b; The MathWorks, Inc.) (Data S2); briefly, individual nuclei were segmented from the DAPI channel using a subroutine from the LivemRNA software package.^100^ Stripes were then automatically identified by the following procedure: (1) bin nuclei by anterior-posterior (AP) coordinate; (2) within each bin, calculate a smoothed fluorescence profile along the dorsoventral (DV) coordinate based on the average fluorescence of each nucleus and its DV position; (3) identify peaks in the fluorescence profile for each bin; (4) align peaks across bins. Within each bin, nuclei falling within the AP coordinates for the half maximum height of a peak (on either side) were automatically considered to belong to the corresponding stripe. Manual curation was applied to fix any errors in stripe identification. Each stripe was then fitted lengthwise (AP axis) with a piecewise linear function through the middle, where for each line segment the stripe width was calculated perpendicular to the segment as the largest distance between the centers of nuclei “belonging” to the segment (i.e., nuclei with AP position falling between the AP coordinates of the two ends of the segment). Overall stripe width was calculated as the average of the widths of constituent segments. For analyzing *tinB* mutant lines, Z-projections of max intensity were generated using ImageJ and then embryos rotated and cropped to the minimum size in which the entire embryo still fitted the image. Composite images were then concatenated together and a montage was made using a scale factor of 1.0. Next, nuclear intensities were measured for each embryo in the montage. Channels were split, and in the DAPI channel the montage was smoothened twice. A threshold was manually set and applied, after which we used the “analyze particles” function based on a selection range of 100 to infinity. This threshold range was overlaid with the reporter channel, and nuclear intensities per embryo were retrieved using the ROI Manager.

#### Motif prediction analysis of random sequences

Position weight matrices (PWMs) for *Drosophila melanogaster* and their logos were obtained from FlyFactorSurvey.^101^ PWMs for specific stages of fly development were retrieved from Li and Wunderlich.^102^ Motif search analysis was done using FIMO^103^ and setting a threshold p-value of 0.001. The top 30% highest PWM-scores were selected to explore putative candidates for TFs binding sites.

#### Information content

Information content for each of the TF motifs can be estimated using the Kullback-Leibler distance:

$$I_{motif}=\sum_{i=1}^{L}\sum_{n=A}^{T}p_{i,n}\;\log_{2}\left(\frac{p_{i,n}}{b_{n}}\right)$$

where $p_{i,n}$ is the probability of observing the nucleotide “n” at position “i” and $b_{n}$ is the background frequency of nucleotide “n”. These values can be an indicative of how frequent a motif hit is expected by chance where $2^{-I_{motif}}$is an approximation of the probability for this event.^43^^,^^104^ The empirical cumulative distribution plot for the information content scores was done in R.

### Quantification and statistical analysis

All statistical details of experiments can be found in the figure legends, figures and/or results, including the statistical tests used, exact value of n and what n represents.

## Data Availability

• Data reported in this paper will be shared by the lead contact upon request. • All original code is available in this paper’s supplemental information. • Any additional information required to reanalyze the data reported in this paper is available from the lead contact upon request.

## Supplemental information

Document S1. Figures S1–S5

Table S1. Sequences of enhancer variants and probes

Data S1. Script-GAC

Data S2. Script-MLP

Document S2. Article plus supplemental information

## References

[1] Carroll S.B. (2008). Evo-devo and an expanding evolutionary synthesis: a genetic theory of morphological evolution. Cell.

[2] Jindal G.A., Farley E.K. (2021). Enhancer grammar in development, evolution, and disease: dependencies and interplay. Dev. Cell.

[3] Monteiro A., Gupta M.D. (2016). Identifying coopted networks and causative mutations in the origin of novel complex traits. Curr. Top. Dev. Biol..

[4] Stern D.L., Orgogozo V. (2008). The loci of evolution: how predictable is genetic evolution?. Evolution.

[5] Majic P., Payne J.L. (2020). Enhancers facilitate the birth of de novo genes and gene integration into regulatory networks. Mol. Biol. Evol..

[6] Nghe P., de Vos M.G.J., Kingma E., Kogenaru M., Poelwijk F.J., Laan L., Tans S.J. (2020). Predicting evolution using regulatory architecture. Annu. Rev. Biophys..

[7] Koshikawa S. (2015). Enhancer modularity and the evolution of new traits. Fly (Austin).

[8] Gompel N., Prud’homme B., Wittkopp P.J., Kassner V.A., Carroll S.B. (2005). Chance caught on the wing: cis-regulatory evolution and the origin of pigment patterns in *Drosophila*. Nature.

[9] Chan Y.F., Marks M.E., Jones F.C., Villarreal G., Shapiro M.D., Brady S.D., Southwick A.M., Absher D.M., Grimwood J., Schmutz J. (2010). Adaptive evolution of pelvic reduction in sticklebacks by recurrent deletion of a Pitx1 enhancer. Science.

[10] Kvon E.Z., Waymack R., Gad M., Wunderlich Z. (2021). Enhancer redundancy in development and disease. Nat. Rev. Genet..

[11] Long H.K., Prescott S.L., Wysocka J. (2016). Ever-changing landscapes: transcriptional enhancers in development and evolution. Cell.

[12] Erwin D.H., Davidson E.H. (2009). The evolution of hierarchical gene regulatory networks. Nat. Rev. Genet..

[13] Emera D., Yin J., Reilly S.K., Gockley J., Noonan J.P. (2016). Origin and evolution of developmental enhancers in the mammalian neocortex. Proc. Natl. Acad. Sci. USA.

[14] Indjeian V.B., Kingman G.A., Jones F.C., Guenther C.A., Grimwood J., Schmutz J., Myers R.M., Kingsley D.M. (2016). Evolving new skeletal traits by cis-regulatory changes in bone morphogenetic proteins. Cell.

[15] Lynch V.J., Leclerc R.D., May G., Wagner G.P. (2011). Transposon-mediated rewiring of gene regulatory networks contributed to the evolution of pregnancy in mammals. Nat. Genet..

[16] Fong S.L., Capra J.A. (2022).

[17] Rebeiz M., Jikomes N., Kassner V.A., Carroll S.B. (2011). Evolutionary origin of a novel gene expression pattern through co-option of the latent activities of existing regulatory sequences. Proc. Natl. Acad. Sci. USA.

[18] Koshikawa S., Giorgianni M.W., Vaccaro K., Kassner V.A., Yoder J.H., Werner T., Carroll S.B. (2015). Gain of cis-regulatory activities underlies novel domains of wingless gene expression in *Drosophila*. Proc. Natl. Acad. Sci. USA.

[19] Arnold C.D., Gerlach D., Spies D., Matts J.A., Sytnikova Y.A., Pagani M., Lau N.C., Stark A. (2014). Quantitative genome-wide enhancer activity maps for five *Drosophila* species show functional enhancer conservation and turnover during cis-regulatory evolution. Nat. Genet..

[20] Smith R.P., Riesenfeld S.J., Holloway A.K., Li Q., Murphy K.K., Feliciano N.M., Orecchia L., Oksenberg N., Pollard K.S., Ahituv N. (2013). A compact, in vivo screen of all 6-mers reveals drivers of tissue-specific expression and guides synthetic regulatory element design. Genome Biol..

[21] Fuqua T., Jordan J., van Breugel M.E., Halavatyi A., Tischer C., Polidoro P., Abe N., Tsai A., Mann R.S., Stern D.L., Crocker J. (2020). Dense and pleiotropic regulatory information in a developmental enhancer. Nature.

[22] Preger-Ben Noon E., Sabarís G., Ortiz D.M., Sager J., Liebowitz A., Stern D.L., Frankel N. (2018). Comprehensive analysis of a cis-regulatory region reveals pleiotropy in enhancer function. Cell Rep..

[23] Frankel N., Davis G.K., Vargas D., Wang S., Payre F., Stern D.L. (2010). Phenotypic robustness conferred by apparently redundant transcriptional enhancers. Nature.

[24] Allan D.W., Thor S. (2015). Transcriptional selectors, masters, and combinatorial codes: regulatory principles of neural subtype specification. Wiley Interdiscip. Rev. Dev. Biol..

[25] Stanojevic D., Small S., Levine M. (1991). Regulation of a segmentation stripe by overlapping activators and repressors in the *Drosophila* embryo. Science.

[26] Small S., Kraut R., Hoey T., Warrior R., Levine M. (1991). Transcriptional regulation of a pair-rule stripe in *Drosophila*. Genes Dev..

[27] Small S., Blair A., Levine M. (1992). Regulation of even-skipped stripe 2 in the *Drosophila* embryo. EMBO J..

[28] Bier E., Jan L.Y., Jan Y.N. (1990). rhomboid, a gene required for dorsoventral axis establishment and peripheral nervous system development in *Drosophila melanogaster*. Genes Dev..

[29] Ip Y.T., Park R.E., Kosman D., Bier E., Levine M. (1992). The dorsal gradient morphogen regulates stripes of rhomboid expression in the presumptive neuroectoderm of the *Drosophila* embryo. Genes Dev..

[30] Markstein M., Zinzen R., Markstein P., Yee K.-P., Erives A., Stathopoulos A., Levine M. (2004). A regulatory code for neurogenic gene expression in the *Drosophila* embryo. Development.

[31] Jiang J., Kosman D., Ip Y.T., Levine M. (1991). The dorsal morphogen gradient regulates the mesoderm determinant twist in early *Drosophila* embryos. Genes Dev..

[32] Pan D.J., Huang J.D., Courey A.J. (1991). Functional analysis of the *Drosophila* twist promoter reveals a dorsal-binding ventral activator region. Genes Dev..

[33] Thisse C., Perrin-Schmitt F., Stoetzel C., Thisse B. (1991). Sequence-specific transactivation of the *Drosophila* twist gene by the dorsal gene product. Cell.

[34] Crocker J., Ilsley G.R. (2017). Using synthetic biology to study gene regulatory evolution. Curr. Opin. Genet. Dev..

[35] Vincent B.J., Estrada J., DePace A.H. (2016). The appeasement of Doug: a synthetic approach to enhancer biology. Integr. Biol. (Camb).

[36] Zaffran S., Reim I., Qian L., Lo P.C., Bodmer R., Frasch M. (2006). Cardioblast-intrinsic Tinman activity controls proper diversification and differentiation of myocardial cells in *Drosophila*. Development.

[37] Yin Z., Xu X.L., Frasch M. (1997). Regulation of the twist target gene tinman by modular cis-regulatory elements during early mesoderm development. Development.

[38] Crocker J., Ilsley G.R., Stern D.L. (2016). Quantitatively predictable control of *Drosophila* transcriptional enhancers in vivo with engineered transcription factors. Nat. Genet..

[39] Beerli R.R., Segal D.J., Dreier B., Barbas C.F. (1998). Toward controlling gene expression at will: specific regulation of the erbB-2/HER-2 promoter by using polydactyl zinc finger proteins constructed from modular building blocks. Proc. Natl. Acad. Sci. USA.

[40] Webster N., Jin J.R., Green S., Hollis M., Chambon P. (1988). The yeast UASG is a transcriptional enhancer in human hela cells in the presence of the GAL4 trans-activator. Cell.

[41] Kakidani H., Ptashne M. (1988). GAL4 activates gene expression in mammalian cells. Cell.

[42] de Boer C.G., Vaishnav E.D., Sadeh R., Abeyta E.L., Friedman N., Regev A. (2019). Deciphering eukaryotic gene-regulatory logic with 100 million random promoters. Nat. Biotechnol..

[43] Wunderlich Z., Mirny L.A. (2009). Different gene regulation strategies revealed by analysis of binding motifs. Trends Genet..

[44] Kvon E.Z., Kazmar T., Stampfel G., Yáñez-Cuna J.O., Pagani M., Schernhuber K., Dickson B.J., Stark A. (2014). Genome-scale functional characterization of *Drosophila* developmental enhancers in vivo. Nature.

[45] de Almeida B.P., Reiter F., Pagani M., Stark A. (2022). DeepSTARR predicts enhancer activity from DNA sequence and enables the de novo design of synthetic enhancers. Nat. Genet..

[46] Klemm S.L., Shipony Z., Greenleaf W.J. (2019). Chromatin accessibility and the regulatory epigenome. Nat. Rev. Genet..

[47] Peng P.C., Khoueiry P., Girardot C., Reddington J.P., Garfield D.A., Furlong E.E.M., Sinha S. (2019). The role of chromatin accessibility in cis-regulatory evolution. Genome Biol. Evol..

[48] Xin Y., le Poul Y., Ling L., Museridze M., Mühling B., Jaenichen R., Osipova E., Gompel N. (2020). Enhancer evolutionary co-option through shared chromatin accessibility input. Proc. Natl. Acad. Sci. USA.

[49] Zaret K.S., Carroll J.S. (2011). Pioneer transcription factors: establishing competence for gene expression. Genes Dev..

[50] Hansen J.L., Loell K.J., Cohen B.A. (2022). The pioneer factor hypothesis is not necessary to explain ectopic liver gene activation. eLife.

[51] Sun Y., Nien C.Y., Chen K., Liu H.Y., Johnston J., Zeitlinger J., Rushlow C. (2015). Zelda overcomes the high intrinsic nucleosome barrier at enhancers during *Drosophila* zygotic genome activation. Genome Res..

[52] Schulz K.N., Bondra E.R., Moshe A., Villalta J.E., Lieb J.D., Kaplan T., McKay D.J., Harrison M.M. (2015). Zelda is differentially required for chromatin accessibility, transcription factor binding, and gene expression in the early *Drosophila* embryo. Genome Res..

[53] Jacobs J., Atkins M., Davie K., Imrichova H., Romanelli L., Christiaens V., Hulselmans G., Potier D., Wouters J., Taskiran I.I. (2018). The transcription factor Grainy head primes epithelial enhancers for spatiotemporal activation by displacing nucleosomes. Nat. Genet..

[54] Foo S.M., Sun Y., Lim B., Ziukaite R., O’Brien K., Nien C.Y., Kirov N., Shvartsman S.Y., Rushlow C.A. (2014). Zelda potentiates morphogen activity by increasing chromatin accessibility. Curr. Biol..

[55] Nevil M., Gibson T.J., Bartolutti C., Iyengar A., Harrison M.M. (2020). Establishment of chromatin accessibility by the conserved transcription factor Grainy head is developmentally regulated. Development.

[56] Harrison M.M., Li X.Y., Kaplan T., Botchan M.R., Eisen M.B. (2011). Zelda Binding in the early *Drosophila melanogaster* embryo marks regions subsequently activated at the maternal-to-zygotic transition. PLoS Genet..

[57] Iwafuchi-Doi M. (2019). The mechanistic basis for chromatin regulation by pioneer transcription factors. Wiley Interdiscip. Rev. Syst. Biol. Med..

[58] Larson E.D., Komori H., Gibson T.J., Ostgaard C.M., Hamm D.C., Schnell J.M., Lee C.Y., Harrison M.M. (2021). Cell-type-specific chromatin occupancy by the pioneer factor Zelda drives key developmental transitions in *Drosophila*. Nat. Commun..

[59] Slattery M., Riley T., Liu P., Abe N., Gomez-Alcala P., Dror I., Zhou T., Rohs R., Honig B., Bussemaker H.J., Mann R.S. (2011). Cofactor binding evokes latent differences in DNA binding specificity between hox proteins. Cell.

[60] Loker R., Sanner J.E., Mann R.S. (2021). Cell-type-specific Hox regulatory strategies orchestrate tissue identity. Curr. Biol..

[61] López-Rivera F., Foster Rhoades O.K., Vincent B.J., Pym E.C.G., Bragdon M.D.J., Estrada J., DePace A.H., Wunderlich Z. (2020). A mutation in the *Drosophila melanogaster* eve stripe 2 minimal enhancer is buffered by flanking sequences. G3 (Bethesda).

[62] Halfon M.S. (2019). Studying transcriptional enhancers: the founder fallacy, validation creep, and other biases. Trends Genet..

[63] Lindhorst D., Halfon M.S. (2022).

[64] Song Y., Park J.O., Tanner L., Nagano Y., Rabinowitz J.D., Shvartsman S.Y. (2019). Energy budget of *Drosophila* embryogenesis. Curr. Biol..

[65] Kvon E.Z., Zhu Y., Kelman G., Novak C.S., Plajzer-Frick I., Kato M., Garvin T.H., Pham Q., Harrington A.N., Hunter R.D. (2020). Comprehensive in vivo interrogation reveals phenotypic impact of human enhancer variants. Cell.

[66] le Poul Y., Xin Y., Ling L., Mühling B., Jaenichen R., Hörl D., Bunk D., Harz H., Leonhardt H., Wang Y. (2020). Regulatory encoding of quantitative variation in spatial activity of a *Drosophila* enhancer. Sci. Adv..

[67] Frankel N.S., Erezyilmaz D.F., McGregor A.P., Wang S., Payre F., Stern D.L. (2011). Morphological evolution caused by many subtle-effect substitutions in regulatory DNA. Nature.

[68] Rebeiz M., Pool J.E., Kassner V.A., Aquadro C.F., Carroll S.B. (2009). Stepwise modification of a modular enhancer underlies adaptation in a *Drosophila* population. Science.

[69] Guerreiro I., Nunes A., Woltering J.M., Casaca A., Nóvoa A., Vinagre T., Hunter M.E., Duboule D., Mallo M. (2013). Role of a polymorphism in a Hox/Pax-responsive enhancer in the evolution of the vertebrate spine. Proc. Natl. Acad. Sci. USA.

[70] Kvon E.Z., Kamneva O.K., Melo U.S., Barozzi I., Osterwalder M., Mannion B.J., Tissières V., Pickle C.S., Plajzer-Frick I., Lee E.A. (2016). Progressive loss of function in a limb enhancer during snake evolution. Cell.

[71] Konishi S., Izawa T., Lin S.Y., Ebana K., Fukuta Y., Sasaki T., Yano M. (2006). An SNP caused loss of seed shattering during rice domestication. Science.

[72] Monroe J.G., Srikant T., Carbonell-Bejerano P., Becker C., Lensink M., Exposito-Alonso M., Klein M., Hildebrandt J., Neumann M., Kliebenstein D. (2022). Mutation bias reflects natural selection in Arabidopsis thaliana. Nature.

[73] Emera D., Wagner G.P. (2012). Transposable element recruitments in the mammalian placenta: impacts and mechanisms. Brief. Funct. Genomics.

[74] Oliver K.R., Greene W.K. (2009). Transposable elements: powerful facilitators of evolution. BioEssays.

[75] Feschotte C. (2008). Transposable elements and the evolution of regulatory networks. Nat. Rev. Genet..

[76] Bourque G., Leong B., Vega V.B., Chen X., Lee Y.L., Srinivasan K.G., Chew J.L., Ruan Y., Wei C.L., Ng H.H., Liu E.T. (2008). Evolution of the mammalian transcription factor binding repertoire via transposable elements. Genome Res..

[77] Gilbertson S.E., Walter H.C., Gardner K., Wren S.N., Vahedi G., Weinmann A.S. (2022). Topologically associating domains are disrupted by evolutionary genome rearrangements forming species-specific enhancer connections in mice and humans. Cell Rep..

[78] Lupiáñez D.G., Spielmann M., Mundlos S. (2016). Breaking TADs: how alterations of chromatin domains result in disease. Trends Genet..

[79] Galupa R., Heard E. (2017). Topologically associating domains in chromosome architecture and gene regulatory landscapes during development, disease, and evolution. Cold Spring Harb. Symp. Quant. Biol..

[80] Eichenlaub M.P., Ettwiller L. (2011). De novo genesis of enhancers in vertebrates. PLoS Biol..

[81] Li S., Hannenhalli S., Ovcharenko I. (2022).

[82] Birnbaum R.Y., Clowney E.J., Agamy O., Kim M.J., Zhao J., Yamanaka T., Pappalardo Z., Clarke S.L., Wenger A.M., Nguyen L. (2012). Coding exons function as tissue-specific enhancers of nearby genes. Genome Res..

[83] Prabhakar S., Visel A., Akiyama J.A., Shoukry M., Lewis K.D., Holt A., Plajzer-Frick I., Morrison H., FitzPatrick D.R., Afzal V. (2008). Human-specific gain of function in a developmental enhancer. Science.

[84] Vaishnav E.D., de Boer C.G., Molinet J., Yassour M., Fan L., Adiconis X., Thompson D.A., Levin J.Z., Cubillos F.A., Regev A. (2022). The evolution, evolvability and engineering of gene regulatory DNA. Nature.

[85] Yona A.H., Alm E.J., Gore J. (2018). Random sequences rapidly evolve into de novo promoters. Nat. Commun..

[86] Surkova S., Golubkova E., Mamon L., Samsonova M. (2018). Dynamic maternal gradients and morphogenetic networks in *Drosophila* early embryo. Biosystems..

[87] Crocker J., Noon E.P.B., Stern D.L. (2016). The soft touch: low-affinity transcription factor binding sites in development and evolution. Curr. Top. Dev. Biol..

[88] Hare E.E., Peterson B.K., Iyer V.N., Meier R., Eisen M.B. (2008). Sepsid even-skipped enhancers are functionally conserved in *Drosophila* despite lack of sequence conservation. PLoS Genet..

[89] Cande J., Goltsev Y., Levine M.S. (2009). Conservation of enhancer location in divergent insects. Proc. Natl. Acad. Sci. USA.

[90] Wade J.T., Grainger D.C. (2018). Spurious transcription and its impact on cell function. Transcription.

[91] Muers M. (2013). Chromatin: evolutionary insights into nucleosomes. Nat. Rev. Genet..

[92] Gophna U. (2018). The unbearable ease of expression—how avoidance of spurious transcription can shape G+C content in bacterial genomes. FEMS Microbiol. Lett..

[93] Laland K.N., Uller T., Feldman M.W., Sterelny K., Müller G.B., Moczek A., Jablonka E., Odling-Smee J. (2015). The extended evolutionary synthesis: its structure, assumptions and predictions. Proc. Biol. Sci..

[94] Kermekchiev M., Pettersson M., Matthias P., Schaffner W. (1991). Every enhancer works with every promoter for all the combinations tested: could new regulatory pathways evolve by enhancer shuffling?. Gene Expr..

[95] Bergman D.T., Jones T.R., Liu V., Ray J., Jagoda E., Siraj L., Kang H.Y., Nasser J., Kane M., Rios A. (2022). Compatibility rules of human enhancer and promoter sequences. Nature.

[96] Li X.C., Fuqua T., van Breugel M.E., Crocker J. (2022).

[97] Lack J.B., Lange J.D., Tang A.D., Corbett-Detig R.B., Pool J.E. (2016). A thousand fly genomes: an expanded *Drosophila* genome nexus. Mol. Biol. Evol..

[98] Fuqua T., Jordan J., Halavatyi A., Tischer C., Richter K., Crocker J. (2021). An open-source semi-automated robotics pipeline for embryo immunohistochemistry. Sci. Rep..

[99] R Core Team (2021).

[100] Garcia H.G., Tikhonov M., Lin A., Gregor T. (2013). Quantitative imaging of transcription in living *Drosophila* embryos links polymerase activity to patterning. Curr. Biol..

[101] Zhu L.J., Christensen R.G., Kazemian M., Hull C.J., Enuameh M.S., Basciotta M.D., Brasefield J.A., Zhu C., Asriyan Y., Lapointe D.S. (2011). FlyFactorSurvey: a database of *Drosophila* transcription factor binding specificities determined using the bacterial one-hybrid system. Nucleic Acids Res..

[102] Li L., Wunderlich Z. (2017). An enhancer’s length and composition are shaped by its regulatory task. Front. Genet..

[103] Grant C.E., Bailey T.L., Noble W.S. (2011). FIMO: scanning for occurrences of a given motif. Bioinformatics.

[104] Schneider T.D., Stormo G.D., Gold L., Ehrenfeucht A. (1986). Information content of binding sites on nucleotide sequences. J. Mol. Biol..

