# Supplementary material for: Enhancer architecture and chromatin accessibility constrain phenotypic space during Drosophila development
Source: Dev Cell. 2023 Jan 9;58(1):51–62.e4. doi: 10.1016/j.devcel.2022.12.003 (PMC9860173; doi:10.1016/j.devcel.2022.12.003)
Supplement: Document S1. Figures S1–S5 [file mmc1.pdf]

Developmental Cell, Volume *58*

Supplemental information

Enhancer architecture and chromatin accessibility constrain phenotypic space during *Drosophila* development

Rafael Galupa, Gilberto Alvarez-Canales, Noa Ottilie Borst, Timothy Fuqua, Lautaro Gandara, Natalia Misunou, Kerstin Richter, Mariana R.P. Alves, Esther Karumbi, Melinda Liu Perkins, Tin Kocijan, Christine A. Rushlow, and Justin Crocker

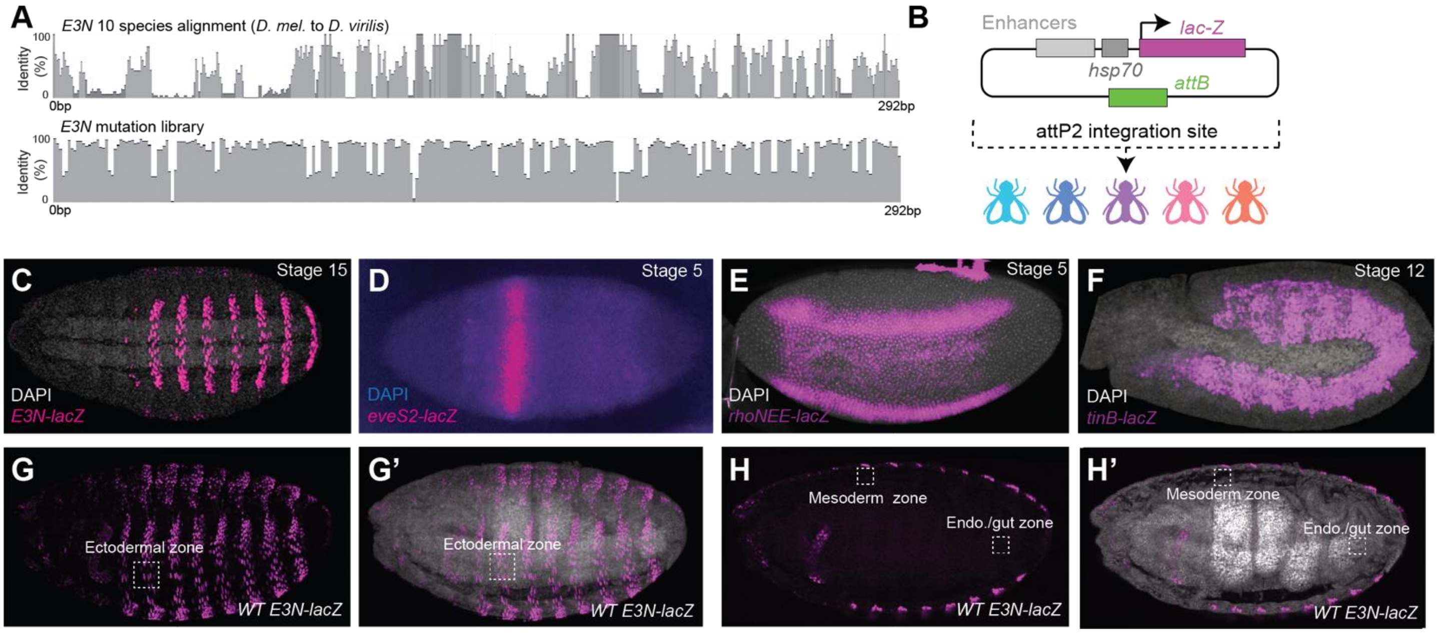

Figure S1. *E3N* library, library construct and analysis of expression patterns in germ layer-related regions, Related to Figures 1-3 and 5. (**A**) Sequence alignment of E3N sequences from 10 Drosophila species (top), and of E3N mutant sequences from the library (bottom). (**B**) Schematic of reporter gene construct used for integration into the *D. melanogaster* genome. (**C-F**) Protein staining of reporter gene expression driven by *E3N* (**C**), *eveS2* (**D**), *rhoNEE* (**E**) and *tinB* (**F**). (**G-H**) Assessment of fluorescence intensity across three different regions of a late-stage embryo, each region associated to a different germ-layer: A2 segment/ectoderm (**G** and **G'**, with DAPI overlay), mesoderm-derived tissue beneath A2 segment (**H** and **H'**, with DAPI overlay) and midgut/endoderm (**H** and **H'**). The same embryo is depicted in (**G**) and (**H**), but at different z-plans.

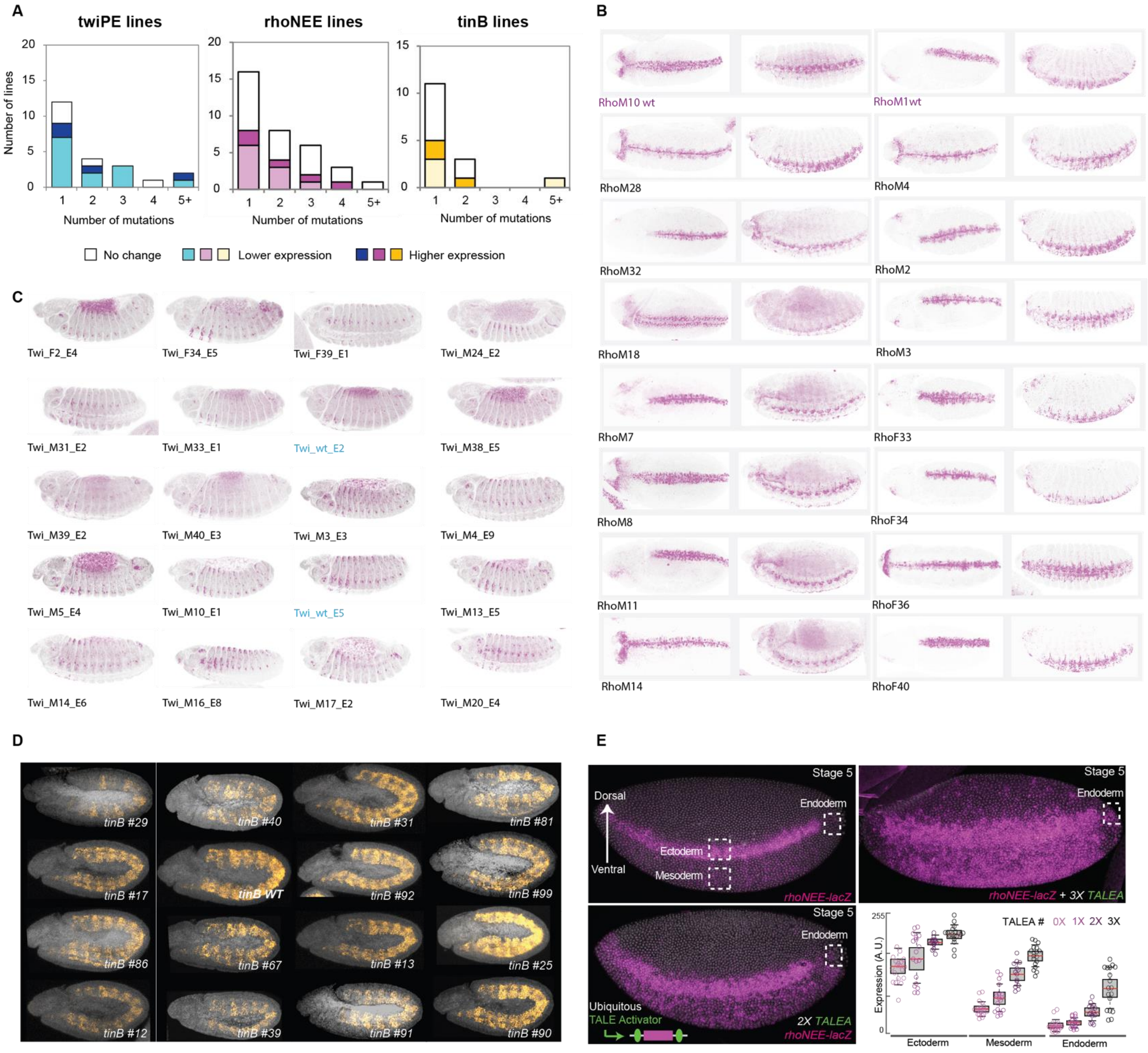

Figure S2. Characterization of early enhancer libraries, Related to Figures 2 and 3. (**A**) Number of mutations per enhancer variant *versus* changes in levels of expression for *twiPE*, *rhoNEE* and *tinB* enhancer lines. (**B**) *rhoNEE* enhancer variants show no evidence for ectopic expression during development; each line is represented at a mid- (left) and late- (right) embryonic stage. (**C**) *twiPE* enhancer variants show no evidence for ectopic expression during late-stage development. (**D**) Examples of stained embryos from different *tinB* mutant variants. (**E**) Extensive activation is required to drive expression outside of native zones of expression. Stage 5 embryos bearing the UAS::TALE-VP64 (TALEA) construct driven by the ubiquitous *nos*::*GAL4* driver and different *rhoNEE-lacZ* constructs, stained for *lacZ* RNA, including wildtype *rhoNEE* (top left) and *rhoNEE* with either two (bottom left) or three (top right) TALEA binding sites. Plots (bottom right) show measurements of the indicated bounding boxes in the top-left panel. Centre line, mean; upper and lower limits, s.d.; whiskers, 95% confidence intervals (CIs).

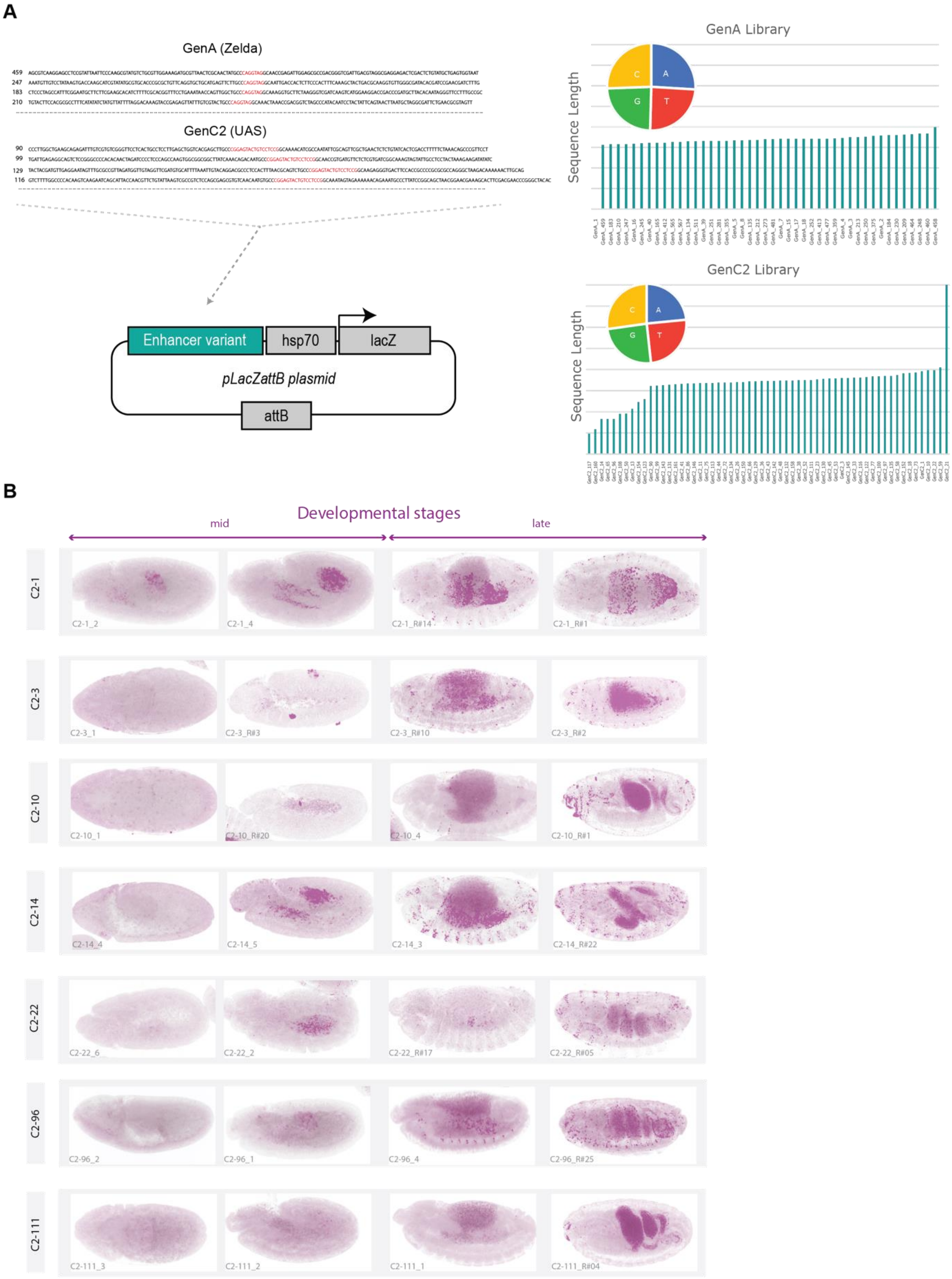

Figure S3. Random DNA libraries, Related to Figures 4 and 5. (**A**) DNA libraries used to explore the regulatory capacity of random DNA. Random DNA containing core motifs (Zelda and UAS are shown for reference) are cloned into the pLacZattB reporter construct (left panel). The sequence lengths and nucleotide compositions are shown along the right panel. (**B**) Random DNA sequences display diverse tissue and cell-type-specific activity patterns; shown are representative embryos across developmental stages. The DNA fragments tested are indicated in the left panels, with the associated stages and embryos across the right panels.

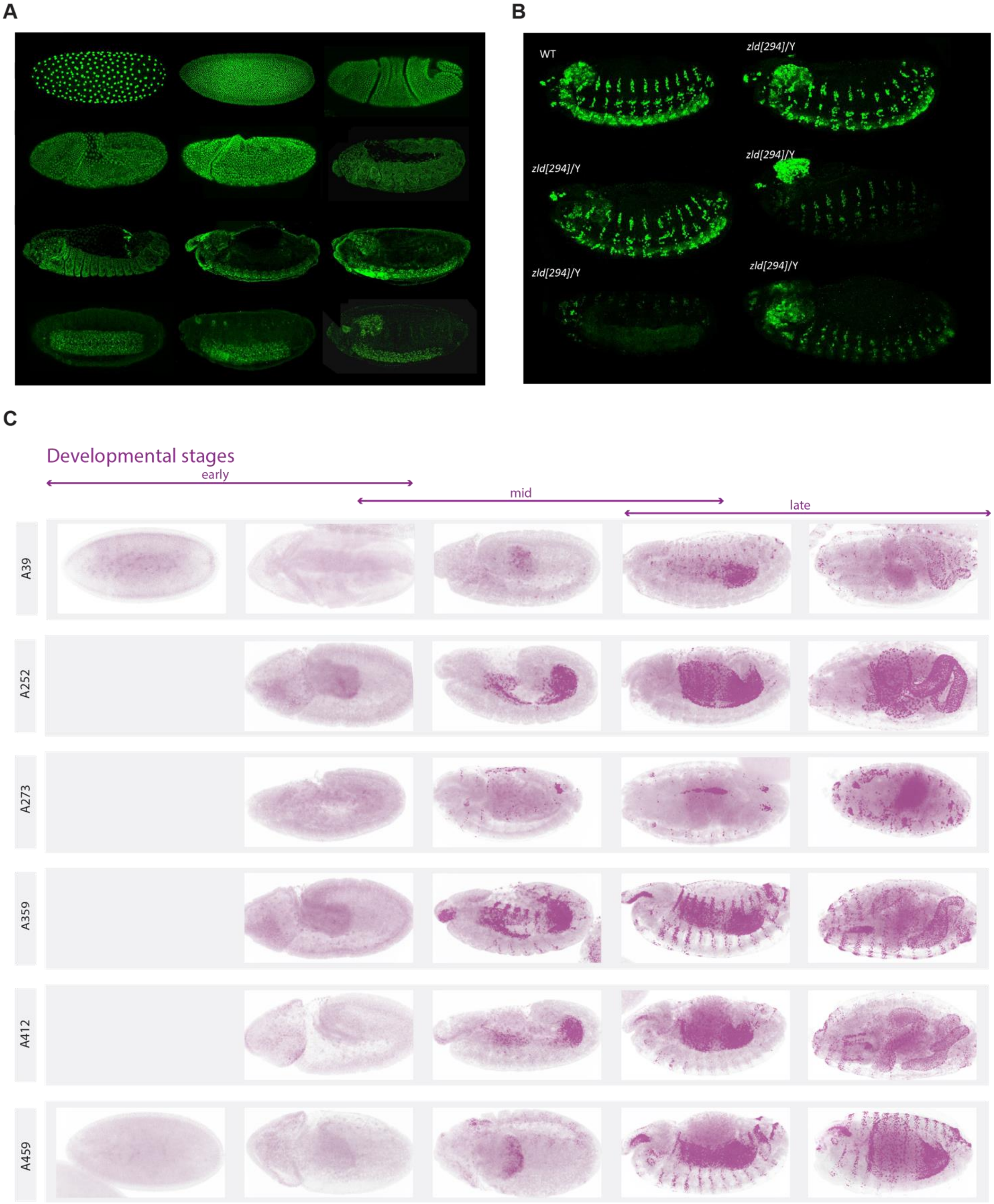

Figure S4. Characterization of Zelda expression, Zelda mutants and Zelda-biased random library, Related to Figure 5. (**A**) Protein staining showing that Zelda is expressed throughout *Drosophila melanogaster* development. (**B**) Late-embryo knockout of Zelda shows phenotypical consequences, namely extensive misregulation of ectodermal derived cell- and tissue-types. (**C**) Random DNA sequences biased with a Zelda motif display diverse tissue and cell-type-specific activity patterns. Shown are representative embryos across developmental stages. The DNA fragments tested are indicated in the left panels, with the associated stages and embryos across the right panels.

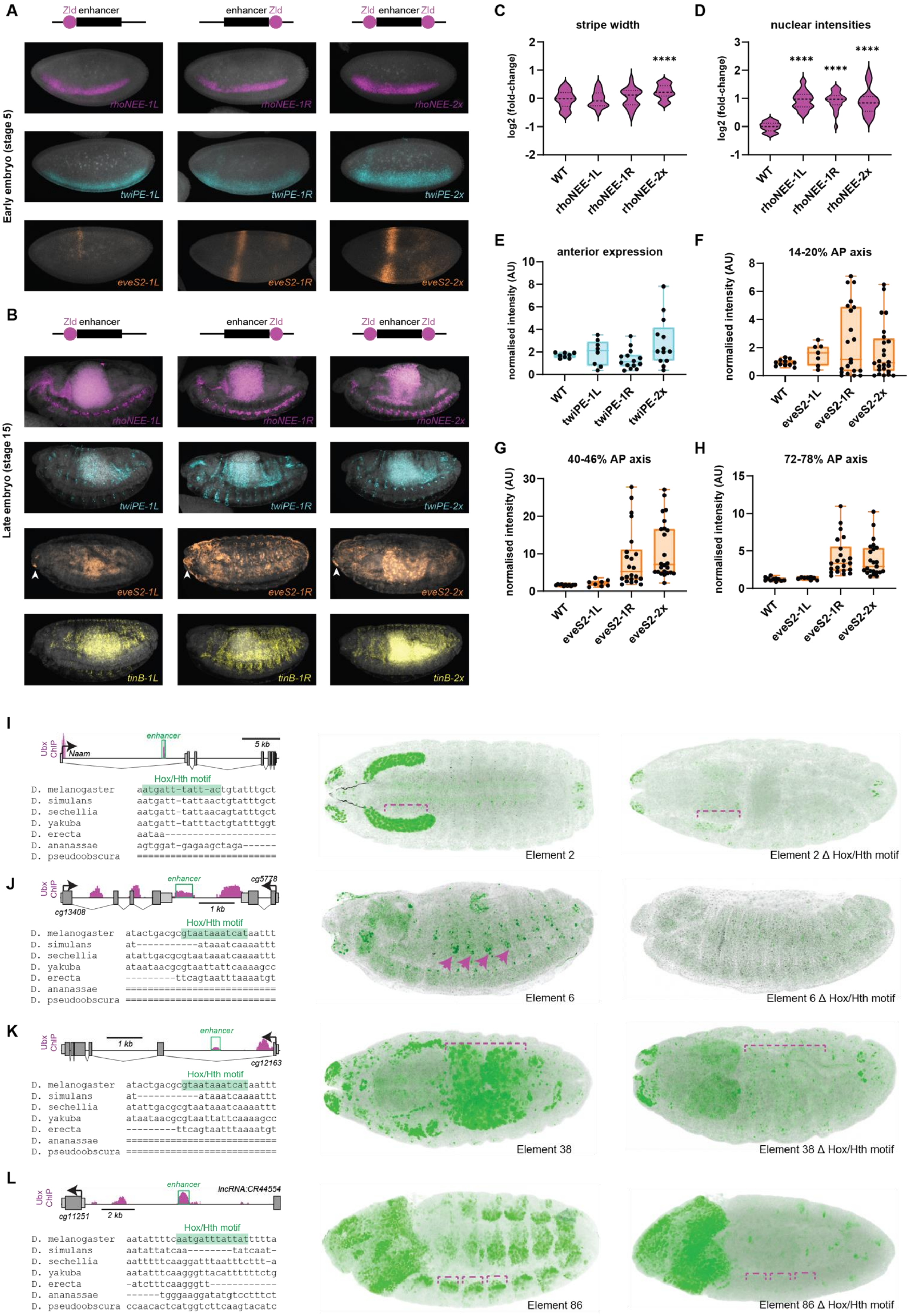

A
Zld enhancer
enhancer Zld
Zld enhancer Zld
Early embryo (stage 5)
rhoNEE-1L
rhoNEE-1R
rhoNEE-2x
twiPE-1L
twiPE-1R
twiPE-2x
eveS2-1L
eveS2-1R
eveS2-2x
B
Late embryo (stage 15)
tinB-1L
tinB-1R
tinB-2x
C
stripe width
log2 (fold-change)
WT
rhoNEE-1L
rhoNEE-1R
rhoNEE-2x
****
D
nuclear intensities
E
anterior expression
normalised intensity (AU)
twiPE-1L
twiPE-1R
twiPE-2x
F
14-20% AP axis
G
40-46% AP axis
H
72-78% AP axis
I
Ubx ChIP
enhancer
5 kb
Naam
Hox/Hth motif
D. melanogaster aatgatt-tatt-actgtatttgct
D. simulans aatgatt-tattaactgtatttgct
D. sechellia aatgatt-tattaacagtatttgct
D. yakuba aatgatt-tatttactgtatttggt
D. erecta aataa--------------------
D. ananassae agtggat-gagaagctaga------
D. pseudoobscura ========================
Element 2
Element 2 Δ Hox/Hth motif
J
cg13408
cg5778
1 kb
D. melanogaster atactgacgcgtaataaatcataattt
D. simulans at-----------ataaatcaaaattt
D. sechellia atattgacgcgtaataaatcaaaattt
D. yakuba ataataacgcgtaattattcaaaagcc
D. erecta ---------ttcagtaatttaaaatgt
D. ananassae ===========================
D. pseudoobscura ===========================
Element 6
Element 6 Δ Hox/Hth motif
K
cg12163
Element 38
Element 38 Δ Hox/Hth motif
L
cg11251
2 kb
lncRNA:CR44554
D. melanogaster aatatttcaatgatttattatttta
D. simulans aatattatcaa--------tatcaat-
D. sechellia aatttttcaaggatttaatttctttt-a
D. yakuba aatatttcaaggggttacatttttttctg
D. erecta -atctttcaagggtt------------
D. ananassae ------tgggaaggatatgtcctttct
D. pseudoobscura ccaacactcatggtcttcaagtacatc
Element 86
Element 86 Δ Hox/Hth motif

Figure S5. Characterization of lines with endogenous enhancer sequences flanked by Zelda motifs and genomic sequences harboring a Ubx/Hth motif, Related to Figure 5. (**A**) Examples of early-stage embryos harboring a *rhoNEE* (top), *twiPE* (middle) or *eveS2* (bottom) enhancer with Zelda motif(s) on the left flank (left), on the right flank (center) or on both flanks (right). (**B**) Examples of late-stage embryos harboring a *rhoNEE* (first line), *twiPE* (second line), *eveS2* (third line) or *tinB* (fourth line) enhancer with Zelda motif(s) on the left flank (left), on the right flank (center) or on both flanks (right). (**C**) Quantification of stripe width for stage5-embryos carrying *rhoNEE* enhancers containing different numbers of ectopic Zelda motifs (****$p<0.0001$, compared to wildtype; two-tailed t-test). (**D**) Quantification of nuclear intensities along the stripes for stage5-embryos carrying *rhoNEE* enhancers containing different numbers of ectopic Zelda motifs (****$p<0.0001$, each compared to wildtype; two-tailed t-test). (**D**) Normalized fluorescence intensities in the anterior region of stage5-embryos carrying *twiPE* enhancers containing different numbers of ectopic Zelda motifs (no statistical significance; two-tailed t-test). (**F-H**) Normalized fluorescence intensities across different regions along the anterior-posterior axis of stage5-embryos carrying *eveS2* enhancers containing different numbers of ectopic Zelda motifs. (**I-L**) Four out of seven genomic sequences harboring a Ubx/Hth motif tested are shown. Left: schematic of genomic region, location of selected sequence, Ubx binding (ChIP-seq) across the locus and sequence conservation across different *Drosophila* species. Center: protein staining of late-stage embryos carrying genomic sequences represented on the left. Right: protein staining of late-stage embryos carrying genomic sequences represented on the left mutated for the Ubx/Hth motif.
